# Psychometric properties of the preschool strengths and difficulties questionnaire (SDQ) in UK 1-to-2-year-olds

**DOI:** 10.1007/s00431-024-05801-2

**Published:** 2024-10-10

**Authors:** Elizabeth M. Byrne, Annette Eneberi, Beth Barker, Ellen Grimas, Jane Iles, Helen Pote, Paul G. Ramchandani, Christine M. O’Farrelly

**Affiliations:** 1 School of Psychology, University of East Anglia, Norfolk, Norwich, NR4 7TJ UK; 2 Faculty of Education, University of Cambridge, Cambridge, UK; 3 Royal Holloway, University of London, London, UK; 4 South London and Maudsley NHS Foundation Trust, London, UK; 5 School of Psychology, University of Surrey, Guildford, UK

**Keywords:** Strengths and Difficulties Questionnaire, Preschool, Emotional and behavioural problems

## Abstract

Early identification of emotional and behavioural difficulties in very young children is crucial for intervention and prevention. The Strengths and Difficulties Questionnaire (SDQ) is a widely used measure of child and adolescent mental health that is brief, cost-effective, and easy to administer. The aims of this study were to establish the validity and reliability of the preschool SDQ in UK 1-2-year-olds. This study investigated the psychometric properties of the Preschool SDQ in a large UK sample (*N* = 2040; female = 46.86%; male = 50.83%, sex not recorded = 2.30%) of infants and toddlers (1-2-year-olds). Analyses were performed at item-level (internal consistency, internal structure, measurement invariance) and scale-level (test–retest reliability, convergent validity). Similar to previous research, confirmatory factor analysis supported a slightly modified five-factor model, including the addition of a positive construal method factor, resulting in satisfactory data fit and a moderately good fitting model. Internal consistency (Cronbach’s alpha) for the SDQ total difficulties score was satisfactory, and higher for externalising over internalising problems. Moderate to strong correlations indicated good test–retest reliability, and moderate correlations indicated convergent validity between the SDQ and Child Behavior Checklist (CBCL), but associations were weaker than those found in studies with older children.

*Conclusions*: The SDQ demonstrated satisfactory psychometric properties, suggesting that it may be a useful tool for the detection of early mental health difficulties, particularly externalising symptoms, even in very young toddlers. Further research is needed to validate the SDQ in younger populations and to establish cut-off scores for clinical interpretation. The implications of these findings are discussed.

**Table:** 

**What is known:** *• Clinically significant emotional and behavioural problems can and do emerge in early toddlerhood.* *• Parenting interventions can reduce mental health problems.* *• Valid, reliable, brief, and affordable tools are needed to identify very young children who may benefit from such support.* **What is new:** *• The Strengths and Difficulties Questionnaire was found to have acceptable factor structure and psychometric properties for use with young children aged 1-2 years old in the UK.* *• The externalising subscale in particular shows promise as an early screening tool.*

**Supplementary Information:**

The online version contains supplementary material available at 10.1007/s00431-024-05801-2.

## Introduction

Emotional and behavioural problems can emerge as early as infancy and toddlerhood [1]. Prevalence rates for 0-5-year-olds are estimated at 16–18%, with approximately half of these children experiencing severe problems [2]. This is broadly aligned with prevalence rates for mental health problems in older children and young people [3, 4]. Whilst transient for many children, these problems can persist [5]. For example, 55% of 1-year-olds with elevated emotional or behavioural problems (> 90th percentile) continued showing elevated scores 1 year later [6]. These enduring difficulties put children at risk of poorer outcomes across the life course [7–10].

With increased acknowledgement of the significance of early mental health, the debate around early identification has followed [11, 12]. Externalising behaviours (e.g. aggression, defiance) are evident as early as 12 months of age [13]. Research suggests that there is an increase in the cumulative onset of these behaviours in 12–17-month-olds [7]. Withdrawn, anxious, and depressed behaviours (i.e. internalising behaviours) have also been observed in children from 12 months of age [14]. There are valid concerns about labelling childhood difficulties as disorders, particularly as behaviours deemed ‘problematic’ in older children often reflect normative development in younger children. However, there is evidence supporting the acceptability, effectiveness, and long-term benefits of early intervention (parenting support) [15]. Intervention success hinges on effective and reliable approaches to identify which children and families may benefit as early as possible. One approach to early identification includes screening children who may be at greater risk of developing enduring emotional or behavioural problems, alongside assessment of the caregiver-child relationship and risk and protective factors [16, 17].

Screening measures that are brief, acceptable to parents and practitioners, accessible (free or low cost), and psychometrically sound are needed [11]. Few measures meet all these criteria. Commonly used parent-report measures include the Child Behavior Checklist (CBCL) [18], Ages and Stages Questionnaire: Social-Emotional, Second Edition (ASQ:SE-2) [19], Infant–Toddler Social-Emotional Assessment (ITSEA) [20], and Brief Infant–Toddler Social-Emotional Assessment (BITSEA) [21]. Each carries costs for administration [16].

The Strengths and Difficulties Questionnaire (SDQ) is another widely used measure of mental health in children (aged 2–17), is briefer in comparison (25 items), and carries no costs for use in public health settings, making it an ideal candidate as a screening tool in community samples [22]. The 4-17-year-old SDQ has good psychometric properties [23–26] and has been shown to be an efficient screening tool for emotional and behavioural problems in preschool children (4-year-olds) [27]. The preschool SDQ (2-4-year-olds) shows encouraging psychometric properties [28–33], with most findings supporting the original five-factor structure proposed by Goodman [23]. Some studies also report evidence for an additional ‘positive construal’ factor whereby positively worded reverse-scored items cross-load onto a positive construal factor [29, 33]. These findings, alongside evidence that the externalising subscale is an accurate and reliable screener for attention-deficit/hyperactivity disorder (ADHD) and disruptive behaviour disorders in primary care settings [34], have driven interest in the SDQ’s potential application in 1-year-olds. Pilot data suggests promising reliability and validity in this younger age group, particularly for externalising behaviours [35].

The present study examines the psychometric properties (internal consistency, factor structure, test–retest reliability, and convergent validity) of the SDQ (2-4-year-old version) in a UK community sample of 1-2-year-olds.

## Methods

### Ethics

The *Healthy Start, Happy Start (HSHS)* study was approved by the NHS Riverside Research Ethics Committee (14/LO/2071) and the Royal Holloway University of London Ethics Committee. Caregivers provided written informed consent. The study was conducted in accordance with the Declaration of Helsinki.

### Participants

Data are derived from the screening phase of the *HSHS* randomised controlled trial [36–38], which investigated the clinical and cost-effectiveness of a brief video-feedback psychological intervention (VIPP-SD) for young children at risk of behaviour problems (ISRCTN58327365).

Data included 2040 children aged 1-2-years-old. Caregivers completed the SDQ between June 2015 and July 2017. See Tables 1 and 2 for child and parent/caregiver characteristics, respectively.

**Table 1:** Child characteristics

	*n*	%
*Total*		
Total screened children	2040	100
Not randomised to trial	1741	85.34
Randomised to trial	299	14.66
*Age*		
Younger (≥ 12 to ≤ 23 months)	1027	50.34
Older (≥ 24 to ≤ 36 months)	1013	49.66
*Sex*		
Male	1037	50.83
Female	956	46.86
Not recorded	47	2.30
*Ethnicity*		
White	1154	56.57
Asian	213	10.44
Black/African/Caribbean	119	5.83
Mixed	79	3.87
Other	43	2.11
Unknown	432	21.18

**Table 2:** Parent/caregiver characteristics

	*n*	*%*
*Age (years)*		
< 20	4	0.20
20–29	358	17.55
30–39	988	48.43
40–49	195	9.56
50 +	7	0.34
Unknown	488	23.92
*Relationship to child*		
Biological mother	1467	71.91
Biological father	162	7.94
Other	13	0.64
Unknown	398	19.51
*Education*		
Pre-GCSE	20	0.98
GCSE	145	7.11
College	414	20.29
Undergraduate	384	18.82
Postgraduate	611	29.95
Unknown	466	22.84

Recruitment took place primarily via health visiting services at children’s developmental reviews, mailshots, or direct approach in clinic waiting rooms and children’s centres. Children were recruited from in and around London, Oxfordshire, Peterborough, and Hertfordshire.

Of the total screened sample, 300 families met the eligibility criteria for the trial and 299 (hereafter the subsample) completed baseline assessments including the CBCL and SDQ. Detailed procedures for the original data collection are described in previous publications from this dataset [36–38].

### Measures

#### Strengths and Difficulties Questionnaire (SDQ): preschool version (2-4-year-old) [39]

The 25-item measure comprises five subscales, each assessed with five questions. Four subscales focus on difficulties related to emotional functioning, peer interaction, conduct, and hyperactivity. Another strengths-based subscale focuses on prosocial behaviours. Individual scales are combined to produce additional scores for internalising difficulties (emotional and peer problems), externalising difficulties (conduct problems and hyperactivity), and total difficulties (internalising and externalising problems). All items are rated on a 3-point Likert scale (0 = *not true*; 1 = *somewhat true*; 2 = *certainly true).* Five positively worded items are reverse-coded. Higher scores reflect greater difficulties for the four difficulties measures and greater prosocial behaviour for the prosocial subscale. The measure has encouraging reliability and validity [28–30, 32, 33, 35].

#### Child behaviour checklist (CBCL)

The CBCL (1.5-to-5-year-olds [40]) is a well-established measure of children’s behavioural and emotional difficulties [18, 40, 41], with good validity for use with children as young as 12 months [42]. It comprises 99 items, each rated on a 3-point Likert scale (0 = *not true*; 1 = *somewhat or sometimes true*; 2 = *very true or often true*). There are eight subscales: emotionally reactive, anxious/depressed, somatic complaints, withdrawn, sleep problems, attention problems, aggressive behaviour, and other problems. Individual scores can be combined, yielding composite scores for internalising problems (emotionally reactive, anxious/depressed, somatic complaints, withdrawn), externalising problems (attentional problems, aggressive behaviour), and total difficulties (all problem scales combined). Higher scores reflect greater difficulties.

### Statistical analysis

Analyses were performed at both item- and scale-level using *R* Statistical Software (v4.2.2) [43].

#### Item-level analysis

##### *Internal structure*

Structural equation modelling (SEM) was conducted, using the *lavaan* [44]* R* package, to evaluate the latent structure of the hypothesized five-factor structure originally proposed by Goodman [39]. Confirmatory factor analysis (CFA) with robust diagonally weighted least squares (DWLS) estimation (designed for ordinal data) modelled five proposed constructs corresponding to the five SDQ subscales. Fit indices included the root mean square error of approximation (RMSEA), Tucker Lewis index (TLI), and comparative fit index (CFI). TLI and CFI values ≥ 0.90 and RMSEA values < 0.08 indicate acceptable model fit, and TLI and CFI values > 0.95 and RMSEA values < 0.05 indicate good fit. Nested models were compared via a likelihood ratio test (scaled *χ*^2^ difference test) and CFI difference.

**Fig. 1:**
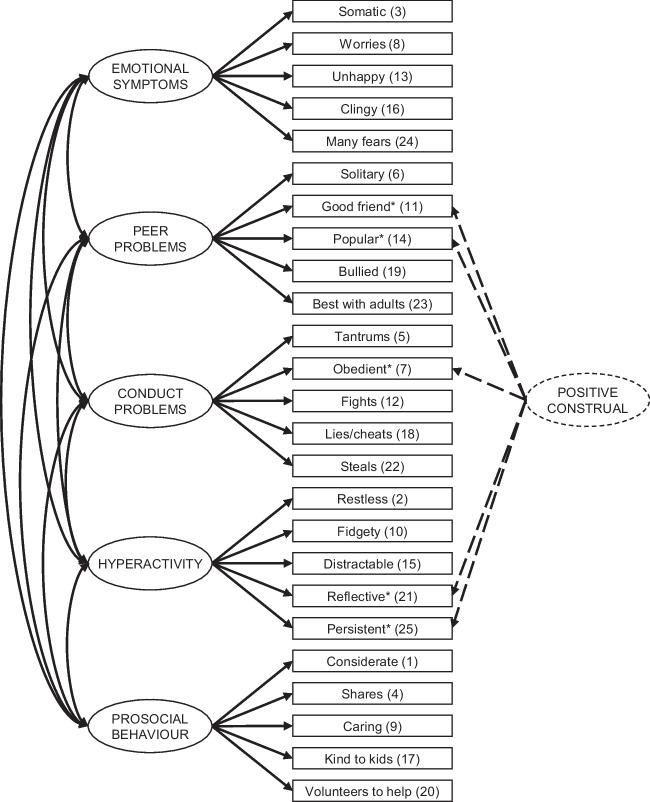
Hypothesised factor structure of the SDQ. Model 1 (M1) is shown by the solid lines and Model 2 (M2) is shown by the solid plus dashed lines. Correlations between the positive construal and SDQ constructs are not shown

Three models were tested (see Fig. 1). Model 1 (M1) indicated the hypothesised five-factor model. Model 2 (M2) tested whether an additional positive construal method factor based on positively worded items (not part of the prosocial construct) explained more variance. Model 3 (M3) tested the winning model, with correlated error terms between observed variables. A data- and theory-driven approach was used to identify potential correlated error terms. The data-driven approach involved identifying pairs of items with significant empirical covariation; modification indices (MI) were computed to find significant sources of improvement in model fit. Covariance paths between within-factor observed variables were included for MI values ≥ 30. The theory-driven approach focused on recognising items conceptually aligned with shared constructs (e.g. semantic similarity). Correlated error terms were added between items reflecting both empirical associations and theoretical similarities to enhance model fit. Adjustments were made incrementally to confirm improvements in model fit (∆ *χ*^2^).

##### Measurement invariance testing

Measurement invariance assessed the psychometric equivalence of constructs across subgroups of children (by age, sex, parental education, ethnicity). Using the winning CFA model, configural, metric, and scalar invariance were tested incrementally (see Supplementary Materials for these results).

##### Internal consistency

Cronbach’s alpha (*α*) and mean inter-item correlation (MIC) indices were calculated for each SDQ subscale using the *psych* [45] and *performance* [46] *R* packages, respectively. *α* ≥ 0.70 is often used as a benchmark of ‘satisfactory’ internal validity; however, there is debate surrounding this cut-off and there is no standard threshold for acceptability [47]. Alphas were interpreted within the context of this study, considering the young age of the sample and the expected dimensionality of the SDQ.

#### Scale-level analysis

##### Test–retest reliability

Spearman’s rank correlation coefficients (Spearman’s rho; *ρ*) were computed to assess test–retest reliability within the subsample separately for younger (1-year-old) and older (2-year-old) children. The interval (days) between the screening and baseline assessment for the subsample was recorded for all children (*M* = 33.87, *SD* = 27.56), 1-year-olds (*M* = 35.11, *SD* = 28.85), and 2-year-olds (*M* = 32.29, *SD* = 25.86).

##### Convergent validity

Convergent validity between SDQ and CBCL was assessed using Spearman’s rank correlations disaggregated by age for younger (1-year-old) and older (2-year-old) children.

#### Missing data

Missing data were dealt with according to the two levels of analysis (see Supplementary Materials for further details).

## Results

### Descriptive statistics

Table 3 provides Time 1 descriptive statistics of the SDQ, including means, SDs, and proportion of missing data. Missing data was generally low, although relatively higher amongst 1-year-olds than 2-year-olds. Time 2 descriptive statistics of the SDQ are provided in Table S1 in the Supplementary Materials.

**Table 3:** Descriptive statistics of the Strengths and Difficulties Questionnaire (SDQ) at Time 1 for the whole sample and by age

SDQ subscale	Potential range	Total (*N* = 2040)			1-year-olds (*n* = 1027)			2-year-olds (*n* = 1013)		
		*n*, missing^a^	M	SD	*n*, missing	M	SD	*N*, missing	M	SD
Emotional symptoms	0–10	11	1.45	1.56	9	1.33	1.50	2	1.58	1.62
Peer problems	0–10	23	2.40	1.80	21	2.60	1.75	2	2.20	1.83
Conduct problems	0–10	24	2.55	1.86	22	2.31	1.67	2	2.78	2.01
Hyperactivity	0–10	14	4.40	2.34	10	4.73	2.31	4	4.07	2.34
Prosocial behaviour	0–10	27	6.22	2.32	25	5.48	2.31	2	6.95	2.10
Internalising problems	0–20	26	3.86	2.74	24	3.93	2.61	2	3.78	2.86
Externalising problems	0–20	29	6.95	3.53	25	7.05	3.31	4	6.85	3.74
Total difficulties	0–40	34	10.80	5.17	30	10.97	4.82	4	10.63	5.50

^a^Missing values were dealt with as per the assessment manual for calculation of subscale scores

Abbreviation: *SDQ* Strengths and Difficulties Questionnaire

### Item-level analysis

#### Internal structure

Table 4 presents the CFA results. Fit statistics for the hypothesized five-factor model (M1) were not satisfactory. The addition of a positive construal factor improved the model fit significantly (∆ *χ*^2^ = 1066, ∆ *df* = 10, *p* < 0.001) and the resulting model was an acceptable fit of the data.

**Table 4:** Confirmatory factor analysis of the Strengths and Difficulties Questionnaire (SDQ): model fit statistics

Model	*Χ*^2^	*df*	Scaling factor	RMSEA	CFI	TLI
M1: Five-factor	4077	265	1.11	0.084	0.69	0.72
M2: Five-factor, positive construal	1254	255	0.94	0.044	0.91	0.93
M3: Five-factor, positive construal, modified^a^	1144	252	0.94	0.042	0.92	0.94

^a^Correlated error terms = items 2 (*restless*) and 10 (*fidgety*), 1 (*considerate*) and 9 (*caring*), and 1 (*considerate*) and 20 (*volunteers*)

Abbreviations*: Χ*^2^ chi-square, *df* degrees of freedom, *RMSEA* root mean square error of approximation, *CFI* comparative fit index, *TLI* Tucker Lewis index

MIs suggested that including several correlated error terms between items would result in significant model improvement; therefore, they were added for items 2 (*restless*) and 10 (*fidgety*) within the hyperactivity construct, and items 1 (*considerate*) and 9 (*caring*), and items 9 and 20 (*helps*), both within the prosocial construct. This modified model (M3) was a significantly better fit of the data than M2 (∆ *χ*^2^ = 120, ∆ *df* = 3, *p* < 0.001) and a moderately good fit overall. Table 5 displays standardised factor loadings of observed variables for all three models. All factor loadings in the winning model (M3) were ≥ 0.50, except for items 11 *(good friend* = 0.43), 21 (*reflective* = 0.37), and 4 (*shares* = 0.48).

**Table 5:** Confirmatory factor analysis: standardised factor loadings

SDQ factors and items^a^	Model		
	M1	M2	M3^c^
*Emotional symptoms*			
Somatic (3)	0.51	0.54	0.54
Worries (8)	0.76	0.77	0.77
Unhappy (13)	0.50	0.50	0.50
Clingy (16)	0.64	0.61	0.61
Many fears (24)	0.67	0.69	0.69
*Peer problems*			
Solitary (6)	0.40	0.57	0.57
Good friend^b^ (11)	0.62	0.40	0.43
Popular^a^ (14)	0.73	0.61	0.64
Bullied (19)	0.25	0.57	0.57
Best with adults (23)	0.33	0.56	0.55
*Conduct problems*			
Tantrums (5)	0.63	0.65	0.65
Obedient^b^ (7)	0.60	0.51	0.53
Fights (12)	0.79	0.76	0.76
Lies/cheats (18)	0.53	0.63	0.62
Steals (22)	0.57	0.65	0.65
*Hyperactivity*			
Restless (2)	0.62	0.71	0.63
Fidgety (10)	0.67	0.77	0.69
Distractible (15)	0.67	0.76	0.78
Reflective^b^ (21)	0.59	0.34	0.37
Persistent^b^ (25)	0.71	0.52	0.55
*Prosocial behaviour*			
Considerate (1)	0.73	0.72	0.67
Shares (4)	0.48	0.47	0.48
Caring (9)	0.76	0.76	0.66
Kind to kids (17)	0.70	0.70	0.71
Volunteers (20)	0.70	0.71	0.67
*Positive construal factor*			
Obedient^b^ (7)		0.54	0.55
Good friend^b^ (11)		0.68	0.69
Popular^b^ (14)		0.64	0.65
Reflective^b^ (21)		0.60	0.59
Persistent^b^ (25)		0.56	0.55

^a^SDQ item numbers in parentheses

^b^Positively worded SDQ items (reversed scored)

^c^Correlated error terms = items 2 (restless) and 10 (fidgety), 1 (considerate) and 9 (caring), and 1 (considerate) and 20 (volunteers)

Abbreviation: SDQ Strengths and Difficulties Questionnaire

Table 6 shows correlations between the five SDQ constructs in M3 and between the prosocial behaviour and positive construal factors. Factor correlation parameter estimates were strongest between the two internalising (emotional symptoms and peer problems = 0.74) and two externalising (conduct problems and hyperactivity = 0.66) constructs. The association between the prosocial behaviour and positive construal factors was also high (− 0.79). The magnitude of these associations suggests considerable conceptual overlap between traits. As would be expected, the lowest correlations were found between the prosocial behaviour factor and constructs of emotional, peer, and conduct problems (all between − 0.11 and − 0.12).

**Table 6:** Factor correlation parameter estimates of the final model (M3)^a^

	1	2	3	4	5	6
1. Emotional symptoms	-					
2. Peer problems	0.74	-				
3. Conduct problems	0.55	0.51	-			
4. Hyperactivity	0.45	0.37	0.66	-		
5. Prosocial behaviour	− 0.11	− 0.11	− 0.12	− 0.33	-	
6. Positive construal factor					− 0.79	-

^a^The positive construal construct was allowed to correlate with the other five constructs of the Strengths and Difficulties Questionnaire (SDQ) but is only shown for the prosocial factor in this table

#### Internal consistency

Cronbach’s *α* and MIC values, indicating the degree of interrelatedness among items within each SDQ dimension, are reported in Table 7. Cronbach’s *α* for the total difficulties score was 0.75 for the total sample, 0.71 in 1-year-olds, and 0.77 in 2-year-olds. Similarly, across all the subscales, alphas were higher for the older versus younger children. Across all groups, alphas were lowest for the peer problems subscale and higher for externalising than internalising problems.

**Table 7:** Cronbach’s alpha (*α*) and mean inter-item correlations (MIC) for each Strengths and Difficulties Questionnaire (SDQ) measure^a^

	Total (*N* = 2040)		1-year-olds (*n* = 1027)		2-year-olds (*n* = 1013)	
SDQ subscale (*n* items)^b^	α	MIC	α	MIC	α	MIC
Emotional symptoms (5)	0.56	0.22	0.54	0.21	0.57	0.21
Peer problems (5)	0.46	0.15	0.42	0.12	0.54	0.19
Conduct problems (5)	0.61	0.25	0.54	0.21	0.66	0.28
Hyperactivity (5)	0.69	0.31	0.66	0.28	0.71	0.32
Prosocial behaviour (5)	0.72	0.34	0.71	0.33	0.70	0.32
Internalising problems (10)	0.61	0.14	0.56	0.13	0.66	0.16
Externalising problems (10)	0.73	0.21	0.70	0.18	0.77	0.25
Total difficulties (20)	0.75	0.13	0.71	0.12	0.79	0.16

^a^Data were imputed using the *mice* package in *R* (Multivariate Imputation by Chained Equations) [48] within each subscale

^b^Total SDQ items = 25

Abbreviations: *α* Cronbach’s alpha, *MIC* mean inter-item correlation, *SDQ* Strengths and Difficulties Questionnaire

### Scale-level analysis

#### Test–retest reliability

Test–retest analyses (correlations) indicated moderate to strong associations between Time 1 and Time 2 across all SDQ subscales for all groups (see Table 8). Correlations ranged from 0.49 to 0.63 for the whole sample, 0.45 to 0.67 for the 1-year-olds, and 0.45 to 0.61 for the 2-year-olds. Correlations for the total difficulties score indicated better test–retest reliability for the older (0.55) versus younger (0.45) children, but associations were mixed for the subscales.

**Table 8:** Test–retest reliability of Strengths and Difficulties Questionnaire (SDQ) (all children and children by age) Spearman’s Rho (*ρ*) correlation coefficients^a^

SDQ subscale	Total (*n* = 299)	1-year-olds (*n* = 167)	2-year-olds (*n* = 132)
Emotional symptoms	0.57	0.52	0.61
Peer problems	0.51	0.46	0.57
Conduct problems	0.63	0.64	0.55
Hyperactivity	0.52	0.52	0.52
Prosocial behaviour	0.61	0.67	0.45
Internalising problems	0.55	0.53	0.59
Externalising problems	0.50	0.45	0.52
Total difficulties	0.49	0.45	0.55

^a^All correlation coefficients were significant (*p* < 0.001). Interval (days): *M* = 33.87, *SD* = 27.56 (total sample); *M* = 35.11, *SD* = 28.85 (1-year-olds); and *M* = 32.29, *SD* = 25.86 (2-year-olds)

Abbreviation:* SDQ* Strengths and Difficulties Questionnaire

#### Convergent validity

The correlation between the SDQ and CBCL total scores for all children who completed both assessments (*n* = 299) was moderate (0.46). There was a stronger relationship between the corresponding subscale measures for externalising (0.52) than internalising problems (0.39). When separating associations by age, a different pattern of results for the younger and older groups of children emerged. As expected, the degree of correspondence between the SDQ and CBCL subscales was higher for the older (2-year-old) than younger (1-year-old) children (correlations between corresponding internalising, externalising, and total problems subscales ranged between 0.54 and 0.58 for 2-year-olds). See Table 9 for a summary of the correlations.

**Table 9:** Convergence (correlations^a^) between the Strengths and Difficulties Questionnaire (SDQ) and Child Behavior Checklist (CBCL)

SDQ subscale	CBCL subscale		
	Internalising problems	Externalising problems	Total problems
*Total children (n* = *299)*			
Emotional symptoms	***0.45***	***0.22***	***0.33***
Peer problems	**0.20**	**0.12**	**0.12**
Conduct problems	***0.31***	***0.47***	***0.43***
Hyperactivity	**0.14**	***0.25***	***0.22***
Prosocial behaviour	0.01	− **0.14**	− 0.07
Internalising problems	***0.39***	**0.20**	***0.27***
Externalising problems	***0.29***	***0.52***	***0.45***
Total difficulties	***0.45***	***0.44***	***0.46***
*1-year-olds (n* = *167)*			
Emotional symptoms	***0.38***	**0.21**	***0.32***
Peer problems	0.11	0.05	0.03
Conduct problems	**0.25**	***0.41***	***0.38***
Hyperactivity	0.13	**0.17**	0.14
Prosocial behaviour	0.05	− 0.05	0.04
Internalising problems	***0.29***	0.13	**0.20**
Externalising problems	***0.29***	***0.44***	***0.39***
Total difficulties	***0.39***	***0.34***	***0.37***
*2-year-olds (n* = *132)*			
Emotional symptoms	***0.48***	**0.22**	***0.30***
Peer problems	***0.38***	**0.28**	***0.32***
Conduct problems	***0.34***	***0.49***	***0.45***
Hyperactivity	**0.18**	***0.37***	***0.32***
Prosocial behaviour	− 0.15	− ***0.36***	− ***0.31***
Internalising problems	***0.54***	***0.31***	***0.38***
Externalising problems	**0.28**	***0.58***	***0.50***
Total difficulties	***0.53***	***0.55***	***0.55***

^a^Bold and *bold italics* denote significant correlations at *p* < 0.05 and *p* < 0.001, respectively

Abbreviation: *SDQ* Strengths and Difficulties Questionnaire, *CBCL* Child Behavior Checklist

## Discussion

This study examined the psychometric properties of the SDQ as a measure of emotional and behavioural problems in a large community sample of 1-to-2-year-olds. Overall, the SDQ shows promise as a cost-effective and brief screening tool for early mental health in young children, particularly for children’s externalising problems.

The original five-factor SDQ model [39] (*emotional symptoms*, *peer problems*, *conduct problems*, *hyperactivity*, *prosocial behaviour*) was a poor fit to the data. However, the addition of a positive construal method factor significantly improved model fit yielding a moderately good fitting model. Whilst inconsistent with previous studies that have found an adequate fit of the original model with pre-schoolers [28, 30, 31], the findings are in line with studies with 2-year-olds and older children (aged 10–19 years) that found better model fit when allowing the positively worded reverse-scored items to cross-load onto a positive construal factor [29, 33, 49, 50]. The findings suggest that while the items do reflect difficulties, much of their associated variance can be attributed to how respondents scored positively worded items.

Model fit was significantly improved by correlating error terms for three pairs of items of similar context, including items 2 and 10 (*restless*, *fidgety*), 1 and 9 (*considerate*, *caring*), and 1 and 20 (*considerate*, *volunteers*). Previous studies have also found that allowing items 2 and 10 (*restless*, *fidgety*) to correlate improves model fit [50–53]. Given the emerging developmental abilities of the sample, the behaviours in these pairs may be difficult for caregivers to distinguish. Adjusting these items for younger children might yield data more sensitive to their developmental stage.

Of the winning model, most factor loadings were ≥ 0.50, indicating that most items effectively represent their corresponding latent factors. However, factor loadings of items 4 (*shares*) in the prosocial subscale, 11 (*good friend*) in the peer problems subscale, and 21 (*reflective*) in the hyperactivity subscale were lower (0.48, 0.43, and 0.37, respectively), meaning they may not effectively capture the attributes they purport to. This corresponds with D’Souza and colleagues’ [29] research involving 2-year-olds and could be due to participants’ young age; 1-2-year-olds are unlikely to have strong peer relationships or higher-order cognitive skills like reflective thinking. This point could also apply to other behaviours, such as *lies/cheats* (item 18), *steals* (item 22), and *bullied* (item 19), which may not be deemed appropriate for such young children. It might be pertinent to modify the items so they better reflect developmentally appropriate abilities/difficulties of younger children [29].

Expectedly, given the downward extension, internal consistency of SDQ subscales was generally weaker than observed in older children (ranging from 2-to-7-year-olds) [28–31]. Still, the overall results align with patterns found in some studies of preschoolers (3-to-6-year-olds) [32, 54]. The total difficulties score met the often-used *α* ≥ 0.70 criteria for satisfactory internal reliability. Overall, scores were higher for externalising over internalising problems in both age groups, consistent with prior accounts of the SDQ’s higher sensitivity to externalising symptoms in younger children [32, 35], possibly due to internalising symptoms being less ‘observable’ in young children than externalising difficulties. Internal consistency was moderate for hyperactivity, prosocial behaviour, and externalising subscales (ranging from 0.66 to 0.77). The emotional, peer, conduct, and internalising problems subscales had lower or unsatisfactory reliability (ranging from 0.42 to 0.61), except for conduct problems in 2-year-olds, which had stronger reliability (0.66). The peer problems subscale had the lowest internal reliability (0.42). Gustafsson and colleagues [55] suggest the prosocial subscale may be less suitable for younger children (aged 1–3 years) given their emerging social skills and peer affiliations.

Moderate to strong correlations indicated good test–retest reliability (*r*s 0.45 to 0.67), but the total difficulties score showed a slightly weaker association compared with prior research with preschool children [34, 55]. Moderate positive correlations were found between equivalent SDQ and CBCL problem subscales in 1-2-year-olds, including internalising (*r* = 0.39), externalising (*r* = 0.52), and total problems (*r* = 0.46), but were weaker than those observed in older children [56]. Overall, stronger associations were found for 2-year-olds than 1-year-olds, particularly for the internalising subscales (1-year-olds *r* = 0.29 versus 2-year-olds *r* = 0.54). Correlations between externalising subscales were also stronger for 2-year-olds (*r* = 0.58) than 1-year-olds (*r* = 0.44), but still moderately strong for both. As internalising and externalising symptoms can overlap more in younger children, those with internalising symptoms may also present with some externalising symptoms [5]. Furthermore, externalising behaviours are likely easier to identify in older children, and thus, parents may be more consistent in their understanding and scoring of similar behaviours across measures. While the relations between broader SDQ subscales (*internalising*, *externalising*, *total*) and corresponding CBCL subscales were encouraging, associations between individual subscales that were summed to provide the broader SDQ subscale scores (*emotional symptoms*, *peer problems*, *conduct problems*, *hyperactivity*) and the CBCL were not as consistent or strong. For example, associations between SDQ hyperactivity and CBCL externalising subscales were 0.17 (1-year-olds), 0.37 (2-year-olds), and 0.25 (all children). This is likely explained by there being fewer items in each individual subscale, meaning they are ‘noisier’ measures of behaviour. That is, each item can introduce more noise into the subscale score, meaning items that might be developmentally inappropriate (e.g. *reflective* within the *hyperactivity* subscale) will have a greater influence on the subscale score. The broader subscales, in contrast, capture behaviour across more items, therefore reducing measurement noise. Patterns of association may also be weaker in younger children as their behaviours are less well developed, and therefore less distinguishable. Their language is also less sophisticated, meaning their views are likely less clearly expressed making it more difficult for parents to rate them. Overall, higher correlations among similar symptoms and divergent patterns amongst less related symptoms are encouraging, providing confidence the SDQ is successfully measuring underlying constructs.

### Limitations

Although children were recruited from the community through routine services, caregivers had a slightly higher graduate-level qualification 52.9%, compared with ~ 40% of people aged 25–34 years in England (based on the 2011 Census). Thus, some caution should be applied when considering sample generalisability. While concurrent validity was assessed via comparisons to the CBCL, data from direct observation or interviews would provide a more robust standard for comparison. However, these types of assessment would be costly, complex to administer, and limited in this age group, whereas the CBCL is a widely used behavioural measure validated for use with 1-2-year-olds [42]. While the positive construal factor was included in the structural equation model to account for a potential method effect of positively worded items, an alternative explanation that the items are an extension of the prosocial behaviour construct cannot be ruled out [29]. There are limitations to cut-off values used to determine goodness-of-fit in SEM. Conventional benchmarks developed for maximum likelihood (ML) estimation with continuous data are debated for their applicability with DWLS estimation (indices are typically better when using DWLS) [57]. Therefore, caution must be exercised and fit indices should be interpreted within the context of children’s age and prior research (i.e. expected result patterns). Previous SDQ psychometric validation studies using the DWLS estimator have used conventional cut-offs [29, 50]. Finally, there are other statistical approaches for psychometric evaluation that could be employed. CFA is an established and robust method widely used to examine the psychometric properties of the SDQ in young children [28–33]. Item response theory (IRT) is another, less commonly used approach [58–60], which offers additional insights into item performance and scale precision across different levels of a latent trait. IRT models describe the probability of a response to a categorical indicator variable in relation to the level of the latent variable [61], indicating item difficulty and respondents’ ability or level of construct endorsement. Whilst beyond the focus and scope of the current paper, future studies using IRT could inform adjustments of SDQ items so that it is more suitable and accurate for use with younger children.

### Implications

The current study provides some evidence for the construct validity (internal structure) of the SDQ as a brief and cost-effective measure of behavioural problems in 1-to-2-year-old children, with psychometric properties comparable to those of slightly older children. Overall, reliability, validity, and consistency measures are better for externalising over internalising symptoms, suggesting the SDQ may be particularly useful for identifying externalising behavioural problems in younger children [35]. This is consistent with Hattangadi and colleagues [34] who found that the externalising, but not internalising, subscale, had sufficient reliability and accuracy to screen 2-4-year-olds (specifically those at risk of ADHD and disruptive behaviour disorders). Focusing on externalising symptoms may be more practically instructive, as they have an earlier onset and show greater stability than internalising problems [5, 62]. Low reliability across some subscales was expected given the young age of participants. For widespread use in measuring children’s behavioural and emotional development, the SDQ might require adjustments to some items for developmental appropriateness. Nevertheless, the findings suggest that the current construction of the SDQ, especially its externalising subscale, might suffice as a useful tool for early screening purposes. However, caution should be exercised if using the tool in its current form in isolation for clinical purposes. Further research is needed to determine the measure’s predictive validity and to assess how it might contribute to screening and early identification pathways. Recommendations for such pathways also include holistic assessments of caregiver-child relationships and consideration of risk and protective factors to determine the potential benefit of monitoring, onward assessment, or early preventative intervention [16]. Ultimately these pathways should be coproduced with families and service providers.

## Conclusion

This study is the first to investigate a large UK sample of infants and toddlers using the SDQ, a widely used questionnaire measure of child and adolescent mental health. By meeting some basic psychometric requirements for reliability and validity, findings indicate that the SDQ could be a useful, brief tool for early detection purposes with children as young as 1 year of age.

## Supplementary information

Below is the link to the electronic supplementary material.

Supplementary file1 (DOCX 31 KB)

## Data Availability

No datasets were generated or analysed during the current study.
