# Supplementary material for: Psychometric properties of the preschool strengths and difficulties questionnaire (SDQ) in UK 1-to-2-year-olds
Source: Eur J Pediatr. 2024 Oct 10;183(12):5339–50. doi: 10.1007/s00431-024-05801-2 (PMC11527971; doi:10.1007/s00431-024-05801-2)
Supplement: Supplementary file 1 — Supplementary file1 (DOCX 31 KB) [file 431_2024_5801_MOESM1_ESM.docx]

**Supplementary materials**

### Descriptive statistics

Table S1. Descriptive statistics of the SDQ at Time 2 for the whole sample and by age.

|  | Total  (*N =* 299) | | | 1-year-olds  (*n =* 167) | | | 2-year-olds  (*n =* 132) | | |
| --- | --- | --- | --- | --- | --- | --- | --- | --- | --- |
| SDQ subscale | *n*  missing | M | SD | *n*  missing | M | SD | *N*  missing | M | SD |
| Emotional symptoms | 1 | 1.49 | 1.52 | 0 | 1.34 | 1.50 | 1 | 1.69 | 1.53 |
| Peer problems | 2 | 3.84 | 1.59 | 1 | 3.83 | 1.54 | 1 | 3.85 | 1.66 |
| Conduct problems | 1 | 3.47 | 2.04 | 0 | 3.02 | 1.90 | 1 | 4.04 | 2.07 |
| Hyperactivity | 1 | 5.95 | 2.04 | 0 | 5.94 | 1.88 | 1 | 5.97 | 2.22 |
| Prosocial behaviour | 2 | 4.75 | 1.77 | 1 | 4.35 | 1.87 | 1 | 5.26 | 1.49 |
| Internalising problems | 2 | 5.33 | 2.41 | 1 | 5.17 | 2.38 | 1 | 5.53 | 2.45 |
| Externalising problems | 1 | 9.42 | 3.28 | 0 | 8.96 | 3.03 | 1 | 10.01 | 3.49 |
| Total difficulties | 2 | 14.76 | 4.46 | 1 | 14.14 | 4.05 | 1 | 15.54 | 4.82 |

*Note.* Missing values were dealt with as per the assessment manual for calculation of subscale scores.

### Measurement invariance

*Measurement invariance analysis*

Configural invariance was assessed by freely estimating all loadings and intercepts across groups, thus testing structural equivalence (i.e., *does the same model structure hold for all groups?*). To test metric invariance (i.e., *are the factor loadings the same between groups?*), factor loadings (slopes) were constrained to be equal across groups, while intercepts were allowed to vary. Finally, factor loadings (slopes) *and* intercepts (means) were constrained to be equal across groups, to measure scalar invariance (i.e., *are the intercepts the same between groups?*). Goodness-of-fit indices were computed for each model, including *χ*^2^, RMSEA, TLI, and CFI. The more constrained model was compared to the less constrained model, incrementally, with ∆ CFI > .010 indicating a *lack* of measurement invariance in the more constrained model. The *R* package, *lavaan*^1^, was used to perform this analysis.

*Measurement invariance results and discussion*

Multi-group models indicated measurement invariance for child sex, ethnicity, and parental/caregiver education (all ∆ CFI <.010). Models also revealed configural and metric, but not scalar (∆ CFI = .011), invariance across child age. Following further investigation (sequentially omitting each item from the model), partial scalar invariance was achieved after omitting item 4 (*shares*). See Table S2 below for a summary of fit indices for all measurement invariance models.

Measurement invariance was found across children’s sex and ethnicity (for the final modified five-factor model), indicating caregivers’ SDQ responses were no different if their child was male or female or if they came from different ethnic backgrounds. Caregivers’ own level of education also did not impact their responses. Full configural and metric invariance, and partial scalar invariance (after omitting item 4 [*shares*] from the prosocial subscale) were found across children’s age (comparing 1- and 2-year-olds), suggesting valid comparisons of the SDQ can be made between children aged 1 and 2 years.^2,3^

Table S2. Goodness-of-fit indices for the measurement invariance analyses (M3) across children’s age, gender, ethnicity, and parental education.

|  | *n* | χ2 | *df* | Scaling  factor | RMSEA | TLI | CFI | ∆ CFI |
| --- | --- | --- | --- | --- | --- | --- | --- | --- |
| *Age* |  |  |  |  |  |  |  |  |
| 1-year-olds | 1027 | 790 | 255 | 1.05 | .045 | .889 | .906 |  |
| 2-year-olds | 1013 | 620 | 255 | 0.94 | .038 | .935 | .945 |  |
| 1. Configural invariance |  | 1418 | 510 | 1.00 | .042 | .913 | .926 |  |
| 2. Metric invariance |  | 1425 | 534 | 1.07 | .040 | .919 | .928 | -.001 |
| 3. Scalar invariance |  | 1586 | 553 | 1.03 | .043 | .909 | .916 | **.011** |
| 4. Partial scalar invariance* |  | 1371 | 505 | 1.01 | .041 | .919 | .926 | .002 |
| *Sex* |  |  |  |  |  |  |  |  |
| Male | 1037 | 714 | 255 | 0.99 | .042 | .919 | .931 |  |
| Female | 956 | 754 | 255 | 1.03 | .045 | .903 | .918 |  |
| 1. Configural invariance |  | 1469 | 510 | 1.01 | .043 | .911 | .925 |  |
| 2. Metric invariance |  | 1442 | 534 | 1.08 | .041 | .920 | .929 | -.004 |
| 3. Scalar invariance |  | 1481 | 553 | 1.04 | .041 | .921 | .927 | .002 |
| *Ethnicity* |  |  |  |  |  |  |  |  |
| White | 1154 | 784 | 255 | 0.98 | .042 | .923 | .935 |  |
| Asian | 213 | 345 | 255 | 1.36 | .041 | .911 | .924 |  |
| Black/African/Caribbean | 119 | 357 | 255 | 1.64 | .058 | .792 | .823 |  |
| 1. Configural invariance |  | 1363 | 765 | 1.35 | .040 | .913 | .926 |  |
| 2. Metric invariance |  | 1378 | 813 | 1.53 | .037 | .922 | .930 | -.004 |
| 3. Scalar invariance |  | 1453 | 851 | 1.46 | .038 | .921 | .925 | .005 |
| *Parental/caregiver education* |  |  |  |  |  |  |  |  |
| Pre-GCSE/GCSE | 165 | 399 | 255 | 1.50 | .059 | .817 | .845 |  |
| College | 414 | 400 | 255 | 1.07 | .037 | .941 | .950 |  |
| UG | 384 | 491 | 255 | 1.21 | .049 | .867 | .887 |  |
| PG | 611 | 501 | 255 | 1.15 | .040 | .929 | .940 |  |
| 1. Configural invariance |  | 1775 | 1020 | 1.24 | .043 | .907 | .921 |  |
| 2. Metric invariance |  | 1820 | 1092 | 1.42 | .041 | .916 | .924 | -.003 |
| 3. Scalar invariance |  | 1913 | 1149 | 1.36 | .041 | .916 | .920 | .004 |

*Note*. *df* = degrees of freedom; RMSEA = root mean square error of approximation; TLI = Tucker Lewis index; CFI = comparative fit index; ∆ CFI = CFI of more constrained model – CFI of less constrained model. **Bold** denotes ∆ CFI > .01 (the cut-off used to determine *lack* of measurement invariance, whereby values < .01 indicate measurement invariance). *Partial scalar invariance was achieved after omitting item 4 (shares).

Measurement invariance was found across children’s sex and ethnicity (for the final modified five-factor model), indicating caregivers’ SDQ responses were no different if their child was male or female or if they came from different ethnic backgrounds. Caregivers’ own level of education also did not impact their responses. Full configural and metric invariance, and partial scalar invariance (after omitting item 4 [*shares*] from the prosocial subscale) were found across children’s age (comparing 1- and 2-year-olds), suggesting valid comparisons of the SDQ can be made between children aged 1 and 2 years.^2,3^

***Missing data***

Across all 25 SDQ items, there were 50,901 valid responses, and 99 missing data points (0.19%), based on the total sample of children (*N*=2,040). Across all CBCL items, there were 29,574 valid responses, and 27 missing data points (0.09%), based on the subsample.

Missing data were dealt with according to the two levels of analysis. For scale-level analyses (test-retest reliability, concurrent validity), missing SDQ data were scaled up pro-rata if ≥3 items were completed (e.g., a score of 4 based on 3 completed items was scaled up to 7 [6.67 rounded up] for 5 items). For the CBCL, as the number of missing values for a given participant did not exceed 8, missing values (*n*=27) were imputed as 0. For item-level analyses (internal consistency, construct validity, measurement invariance testing), missing data were imputed using the *mice* (multivariate imputation by chained equations)^4^ *R* package. Only data from the SDQ were used for item-level analyses, therefore data from the CBCL were not imputed using this approach.

***References for the supplementary materials***

1. Rosseel Y. lavaan: An R Package for Structural Equation Modeling. *J Stat Softw*. 2012;48:1-36. doi:10.18637/jss.v048.i02

2. D’Souza S, Waldie KE, Peterson ER, Underwood L, Morton SMB. Psychometric Properties and Normative Data for the Preschool Strengths and Difficulties Questionnaire in Two-Year-Old Children. *J Abnorm Child Psychol*. 2017;45(2):345-357. doi:10.1007/s10802-016-0176-2

3. Steenkamp JBEM, Baumgartner H. Assessing measurement invariance in cross-national consumer research. *J Consum Res*. 1998;25:78-90. doi:10.1086/209528

4. Buuren S van, Groothuis-Oudshoorn K. mice: Multivariate Imputation by Chained Equations in R. *J Stat Softw*. 2011;45:1-67. doi:10.18637/jss.v045.i03
